# NADPH Oxidase 3 Deficiency Protects From Noise-Induced Sensorineural Hearing Loss

**DOI:** 10.3389/fcell.2022.832314

**Published:** 2022-02-22

**Authors:** Francis Rousset, German Nacher-Soler, Vivianne Beatrix Christina Kokje, Stéphanie Sgroi, Marta Coelho, Karl-Heinz Krause, Pascal Senn

**Affiliations:** ^1^ The Inner Ear and Olfaction Lab, Department of Pathology and Immunology, Faculty of Medicine, University of Geneva, Geneva, Switzerland; ^2^ Department of Clinical Neurosciences, Service of ORL and Head and Neck Surgery, University Hospital of Geneva, Geneva, Switzerland; ^3^ Department of Pathology and Immunology, Faculty of Medicine, University of Geneva, Geneva, Switzerland

**Keywords:** NADPH oxidase, NOX3, cochlea, noise-induced hearing loss, auditory neurons, neuroprotection

## Abstract

The reactive oxygen species (ROS)-generating NADPH oxidase NOX3 isoform is highly and specifically expressed in the inner ear. NOX3 is needed for normal vestibular development but NOX-derived ROS have also been implicated in the pathophysiology of sensorineural hearing loss. The role of NOX-derived ROS in noise-induced hearing loss, however, remains unclear and was addressed with the present study. Two different mouse strains, deficient in NOX3 or its critical subunit p22^phox^, were subjected to a single noise exposure of 2 h using an 8–16 kHz band noise at an intensity of 116–120 decibel sound pressure level. In the hours following noise exposure, there was a significant increase in cochlear mRNA expression of NOX3 in wild type animals. By using RNAscope *in situ* hybridization, NOX3 expression was primarily found in the Rosenthal canal area, colocalizing with auditory neurons. One day after the noise trauma, we observed a high frequency hearing loss in both knock-out mice, as well as their wild type littermates. At day seven after noise trauma however, NOX3 and p22^phox^ knockout mice showed a significantly improved hearing recovery and a marked preservation of neurosensory cochlear structures compared to their wild type littermates. Based on these findings, an active role of NOX3 in the pathophysiology of noise-induced hearing loss can be demonstrated, in line with recent evidence obtained in other forms of acquired hearing loss. The present data demonstrates that the absence of functional NOX3 enhances the hearing recovery phase following noise trauma. This opens an interesting clinical window for pharmacological or molecular intervention aiming at post prevention of noise-induced hearing loss.

**GRAPHICAL ABSTRACT:**
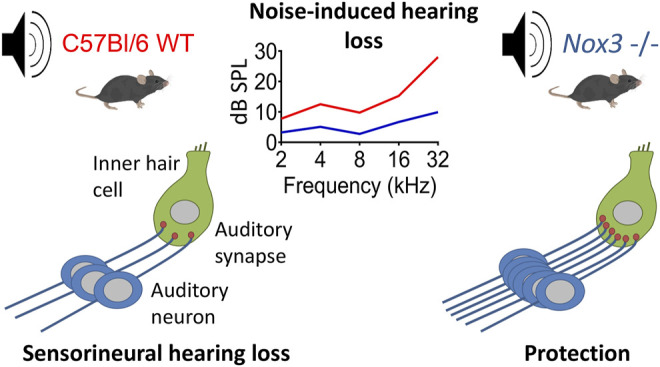


## Introduction

Hearing loss is the most common neurosensory deficit in humans and the third most prevalent chronic disability over 65 years old, affecting over 460 million people world-wide (World Health Organization, 2022). Noise exposure is reported to account for ∼30% of hearing loss cases (Le et al., 2017) and the prevalence of noise-induced hearing loss in industrial populations ranges from 37–60%. Sound pressure vibrations travel to the cochlea and activate inner hair cells at frequency specific locations, leading to glutamate release in the auditory synapses and generation of electrical stimuli traveling from the auditory neurons to the central nervous system, where they are perceived as sound (Wang and Puel, 2018). A variety of insults can damage the peripheral auditory system and lead to transient or permanent hearing loss. Recent evidence suggests that the auditory neurons and their synaptic connections to the hair cells are the most vulnerable elements in the peripheral auditory system, at least for noise-induced hearing loss and presbyacusis (Liberman and Kujawa, 2017; Wu et al., 2019; Rousset et al., 2020; Peineau et al., 2021). Even moderate sound pressure levels over prolonged time periods can cause degeneration of spiral ganglion neurons and their synaptic connections (Kujawa and Liberman, 2006; Kaur et al., 2019). This so-called cochlear synapthopathy can even occur in cochleae with intact hair cell populations and normal audiograms, leading to a situation referred to as hidden hearing loss (Kujawa and Liberman, 2009; Wu et al., 2019). Sensorineural hearing loss persisting beyond few days after any insult, including neuronal apoptosis, is irreversible in humans (Rousset F et al., 2020).

Over the past decades it became clear that oxidative stress is a common denominator of many forms of acquired sensorineural hearing loss (Henderson et al., 2006; Rousset et al., 2015; Fetoni et al., 2019; Ramkumar et al., 2021). Noise-induced oxidative damages, including hair cell membrane lipid peroxidation or DNA oxidation, appear as early as a few hours following the insult (Maulucci et al., 2014). In addition to oxidative stress, specific redox signaling was proposed to lead to excitotoxic degeneration of afferent auditory neurons, as recently demonstrated in a mouse model of age-related hearing loss (Rousset et al., 2020).

Growing evidence suggests that the reactive oxygen species (ROS) generating NADPH oxidase enzyme NOX3 is an important mediator of oxidative damages and a possible common mediator in several forms of sensorineural hearing loss (Mukherjea et al., 2010; Rousset et al., 2015; Rousset et al., 2020; Mohri et al., 2021). NADPH oxidases (NOX) are a family of seven isoenzymes expressed in mammals dedicated to the production of ROS. NOX are virtually expressed in all tissues of the body and have essential physiological functions, including bacteria killing, thyroid hormone synthesis, otoconia formation and redox cellular signaling (Egea et al., 2018). The NOX3 isoform is highly and specifically expressed in the inner ear and needed for normal vestibular development. The physiological role of NOX3 has been demonstrated in NOX3 mutant mice (also referred to as head-tilt mice) where it plays a key role in the formation of otoconia: small bio-crystals essential for the perception of linear accelerations and gravity (Paffenholz et al., 2004). Mice with a loss of function mutation in the common NOX1, NOX2, NOX3, and NOX4 subunit p22^phox^, have a similar vestibular phenotype, comparable to the head-tilt phenotype of NOX3 mutant mice (Nakano et al., 2008). Although NOX3 expression is also found at significant levels in the cochlear part of the inner ear, a clear physiological role in cochlear development or maintenance has not been demonstrated (Bánfi et al., 2004; Rousset et al., 2020) and NOX3 deficient mice exhibit comparable hearing thresholds to wild-type (WT) mice (Nakano et al., 2008; Lavinsky et al., 2015). However, in a pathogenic context such as ageing or cisplatin exposure, the NOX3-derived ROS contribute significantly to morphological and functional consequences with destruction of the neurosensory cells in the cochlea and hearing loss (Rousset et al., 2015). In these two forms of acquired sensorineural hearing loss, NOX3 deficiency was shown to be protective (Mukherjea et al., 2010; Rousset et al., 2020). Noise induced hearing loss is thought to involve similar ROS-induced pathways (Henderson et al., 2006; Maulucci et al., 2014; Dhukhwa et al., 2019; Mohri et al., 2021). Counter-intuitively however, one recent report suggests a protective role of NOX3 in noise-induced hearing loss (Lavinsky et al., 2015). Therefore, clarification of the role of NOX3 in noise-induced hearing loss is needed and our study aims at filling this gap.

In the present study we addressed the role of NOX3 in noise-induced hearing loss by subjecting two different NOX3 deficient mouse strains and their wild type littermates to white noise exposure. One mouse strain carries a loss of function mutation in NOX3 (C57BL/6J-*Nox3* ^*het−4J*^/J) (Flaherty et al., 2011) and the second strain has a constitutive p22^phox^ knockout (C57BL/N *Cyba* knockout) (Pircalabioru et al., 2016). The p22^phox^ knockout strain is devoid of NOX1, NOX2, NOX3 and NOX4 activity (Nakano et al., 2007; Prior et al., 2016). Our results demonstrate that NOX3 is predominantly expressed in the auditory neurons area and is upregulated upon noise exposure. Both NOX3 deficient mouse strains were partially, but significantly protected against noise-induced high frequency hearing loss, concurring with the tonotopic pattern of NOX3 expression in the auditory neurons. Histologically, NOX3 deficient strains showed a significant preservation of cellular structures in the sensory epithelium, the auditory synapses and the spiral ganglion in comparison to their wild type littermates. Based on these findings, an active role of the ROS generating enzyme NOX3 in the pathophysiology of noise-induced hearing loss can be demonstrated, in line with NOX3 implication in other forms of acquired hearing loss such as age-related hearing loss and cisplatin-induced hearing loss. The exclusive and specific expression of NOX3 in the inner ear opens interesting opportunities for pharmacological (Augsburger et al., 2019) or molecular (Rousset et al., 2019) interventions aiming at prevention of several acquired forms of hearing loss including noise-induced hearing loss.

## Results

### NOX3 is Specifically Expressed in Spiral Ganglion Neurons

**FIGURE 1:**
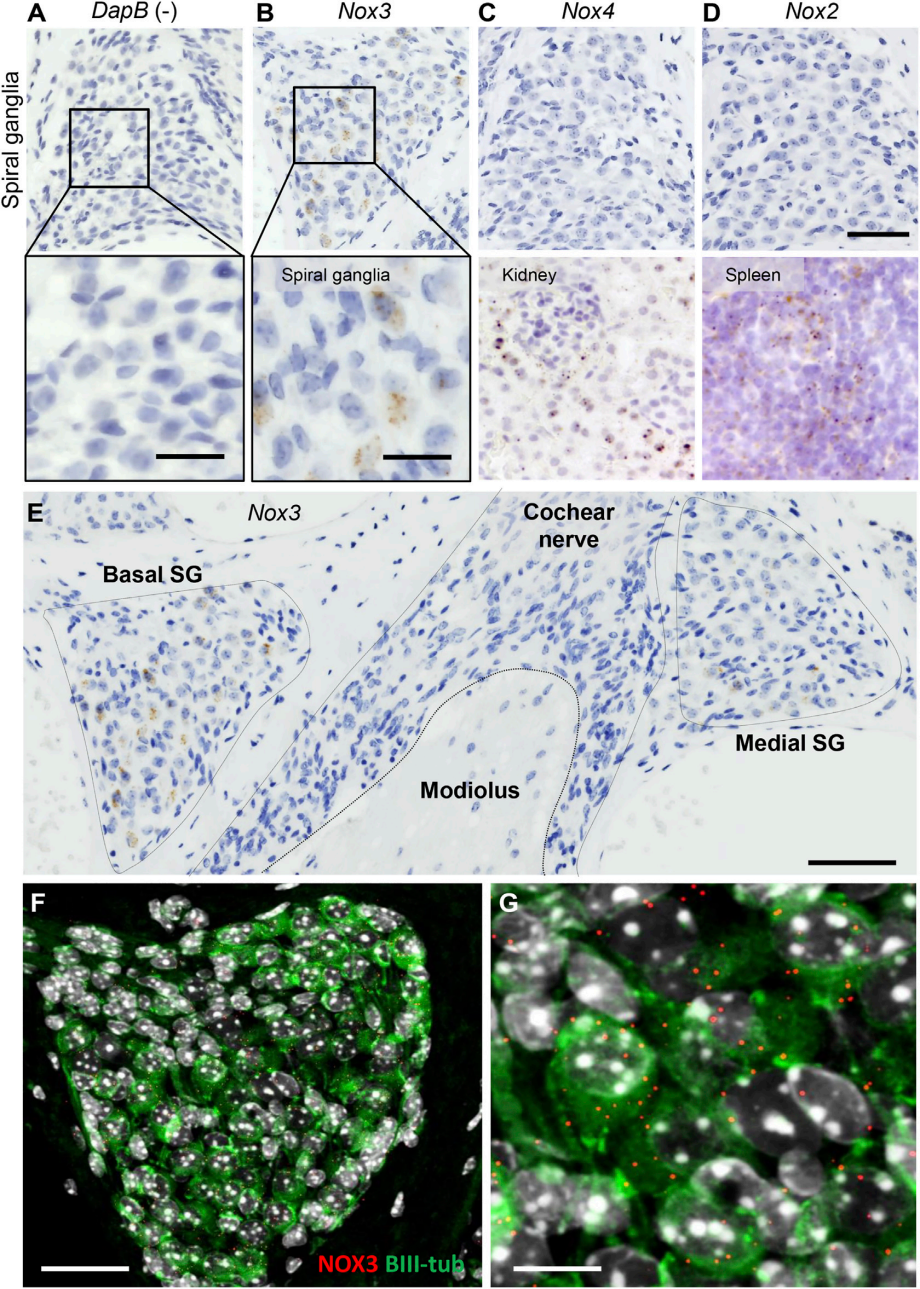
Localization of NOX isoform in the mouse cochlea. RNAscope *in situ* hybridization of *Nox3* **(B)**, *Nox4* (**C**, upper panel) and *Nox2* (**D**, upper panel) in mouse spiral ganglion. Dihydrodipicolinate reductase (*Dapb*) expressed in the bacteria *E. Coli* was used as negative control **(A)**. Scale bar 50 µm (upper panel). **(A, B)** Lower panel show higher magnification of mouse spiral ganglion. Scale bar = 20 µm. **(C)** Kidney and **(D)** spleen slices were respectively used as positive control for *Nox4* and *Nox2*. **(E)** Mid-modiolar view of *Nox3* expression as detected by RNAscope *in situ hybridization* in a mouse cochlea: Nox3 is predominantly observed in the Rosenthal canal but not the central part of the cochlear nerve. scale bar = 100 µm. **(F, G)** Mid modiolar view of the mouse Rosenthal canal immunostained with BIII-tubulin (green) and Nox3 RNAscope fluorescent probe (red dots). Samples were counterstained with DAPI (grey). Scale bars = 50 and 20 µm respectively. Pictures are representative from 6 independent experiments (*n* = 6 WT mice).

NOX-derived reactive oxygen species are prospective contributors of noise-induced damage to the inner ear. Several NOX isoforms, including NOX2, NOX3 and NOX4 were found to be expressed in the mouse and human cochlea (Rousset et al., 2020). Addressing more specifically the localization of NOX in the sub-compartments of the cochlea, we performed a RNAscope *in situ* hybridization (Figure 1; Supplementary Figure S1). As expected, the bacterial gene *DapB*, used as negative control, did not result in any signal (Figure 1A). However, we observed a strong expression of *Nox3* in the spiral ganglion (Figure 1B). Conversely, *Nox2* and *Nox4*, respectively well expressed in kidney and spleen slices, used as positive controls, could not be detected in the spiral ganglion (Figures 1C,D). In the stria vascularis, little Nox3 and Nox4 expression could be found; however, no significant NOX expression was detected in the organ of Corti (Supplementary Figure S1). Interestingly, Nox3 expression was specific from the Rosenthal canal area but no expression could be detected in the central part of the auditory nerve (Figure 1E). By using fluorescent Nox3 probes and BIII tubulin staining (Figures 1F,G), we could observe an important Nox3 expression in the peripheral auditory neurons.

### NOX3 Expression is Induced Upon Noise Exposure

**FIGURE 2:**
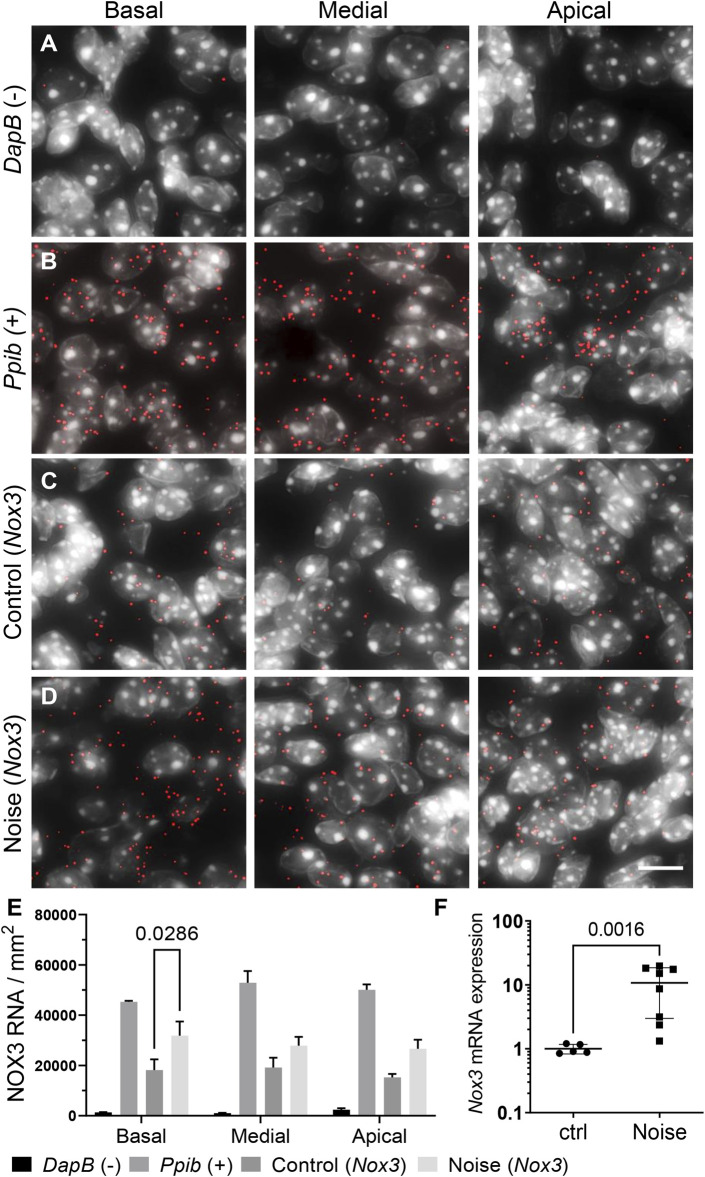
Effect of noise exposure on cochlear expression of NOX3. C57Bl6/J mice were exposed to an 8–16 kHz 116dB SPL noise band for 2 h. 24 h following the noise exposure, cochleae were harvested for *Nox3* RNAscope *in situ* hybridization and Real time qPCR. **(A–D)** RNAscope *in situ* hybridization of dihydrodipicolinate reductase (*Dapb*) expressed in the bacteria *E. Coli* (negative control) **(A)**, Peptidyl-Prolyl Cis-Trans Isomerase B (*Ppib* gene) (positive control) **(B)** and *Nox3* in the mouse Rosenthal canal **(C, D)**. RNAscope signal is visualized with the red dots; all slices were counter stained with DAPI (grey). **(C)** Representative *Nox3* mRNA fluorescent staining in the 3 cochlear turns of non-exposed animals (control). **(D)** Representative *Nox3* mRNA fluorescent staining in the 3 cochlear turns of noise-exposed animals (Noise). Scale bar = 10 µm. Note that lower magnified pictures are available in Supplementary Figure S2. **(E)** Bar graph shows relative mRNA expression level of *Nox3* as quantified from *Nox3* RNAscope signal in the Rosenthal canal before and following noise exposure. *n* = 3 animals/group. *p* = 0.0286 with two-way ANOVA. **(F)** Real time qPCR of *Nox3* mRNA in the whole mouse cochlea. Post noise exposure level of *Nox3* (*n* = 8) was compared to *Nox3* expression in the cochlea of 5 non-exposed animals. *p* = 0.0016 with Mann-Whitney non-parametric test.

Noise over-exposure is known to increase the level of ROS within the cochlea (Maulucci et al., 2014; Haryuna et al., 2016). Therefore, we assessed the expression level of the underlying ROS generating NOX enzymes (Figure 2; Supplementary Figure S2, S3). To compare the expression of NOX3 before and after noise exposure, we used both RNAscope *in situ* hybridization and RT-qPCR (Figure 2). As already shown (Figure 1), *Nox3* mRNA was broadly expressed in the Rosenthal canal (Figure 2C). *Dapb* and *Ppib* genes were respectively used as negative and positive controls (Figures 2A,B; Supplementary Figure S2). Noise exposure resulted in an increase in the number of *Nox3* related punctate dots, each revealing a pool of *Nox3* mRNA (Figures 2D,E; Supplementary Figure S2). The increase in *Nox3* expression was statistically significant in the basal turn of the Rosenthal canal, with a similar trend observed in the medial and apical cochlear turns. The noise-induced upregulation of *Nox3* mRNA was also detected using RT-qPCR from whole cochlear samples (Figure 2F). Note that *Nox3* mRNA expression was neither induced in stria vascularis nor in the organ of Corti, where it remained at low level (Supplementary Figure S1; not shown). Similarly, the expression level of other Nox isoforms, including NOX2, NOX4 and pan-NOX subunit p22^phox^ (*Cyba*) where not significantly affected by the noise stimulus (Supplementary Figure S3). Taken together, these findings demonstrate that NOX3 is the main isoform expressed in auditory neurons and is induced upon ototoxic noise exposure.

### NOX3-Deficient Mice are Partially but Significantly Protected from Noise Induced Hearing Loss

**FIGURE 3:**
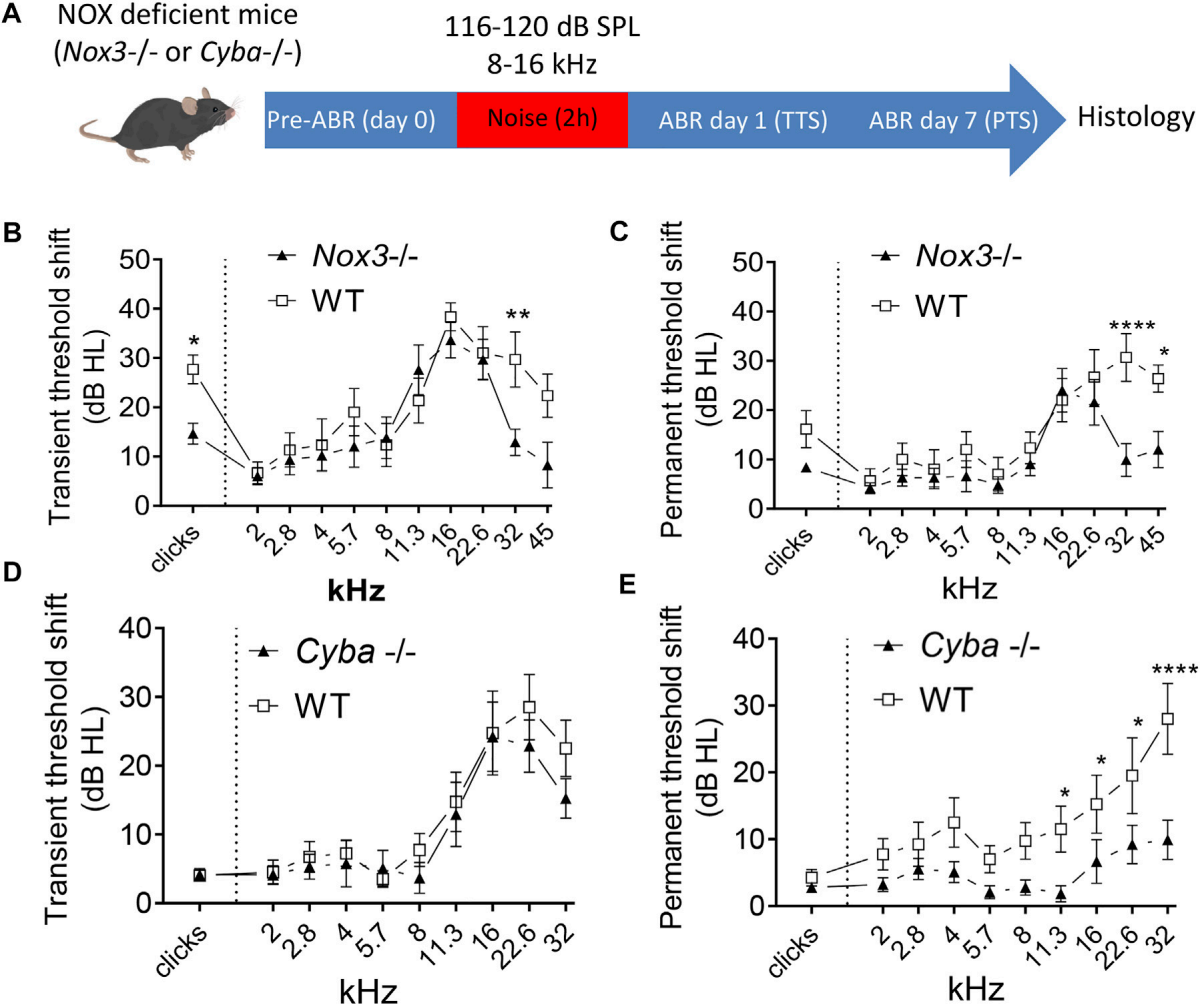
Audiogram of NOX3 deficient mice upon noise exposure. **(A)** Six weeks old NOX3 mutant and p22^phox^ knockout mice and their respective WT and heterozygous littermates were subjected to a first ABR measurement (pre-ABR; Day 0) followed by 8–16 kHz noise band exposure of 2 h duration at 116 dB SPL (for NOX3) or 120 dB SPL (for p22^phox^). Transient threshold shift (TTS) and permanent threshold shifts (PTS) were respectively calculated by substraction of the reference hearing threshold values (preABR; D0) to the D1 and D7 hearing threshold values. **(B, D)** TTS as recorded at day 1 following noise exposure in NOX3 mutant mice **(B)** and p22^phox^ ko mice **(D)** with their respective WT littermates. **(C, E)** PTS as recorded at day 7 following noise exposure in NOX3 mutant mice **(C)** and p22^phox^ ko mice **(E)** with their respective WT littermates. **(B, C)** *n* = 9 *Nox3* ^+/+^; 10 *Nox3* ^−/−^; **(D, E)** *n* = 12 *Cyba*+/+; 13 *Cyba* −/−. To facilitate comparison between WT and NOX3-deficient animals, hearing threshold shifts from heterozygous animals are not shown here but are available in Supplementary Figure S5.

To address whether NOX3 could contribute to noise-induced damages, we assessed the hearing of constitutive NOX3 deficient mice - namely NOX3 and p22^phox^ knockout - following noise exposure (Figure 3, Supplementary Figure S4, S5). At 6 weeks of age, audiograms of NOX3 mutant or p22^phox^ knockout mice were not significantly different from their respective heterozygous and WT littermates (Figures 3B,C, Supplementary Figure S5). Note that NOX3 and p22^phox^ mouse lines were respectively generated in C57Bl6/J and C57Bl6/N genetic backgrounds. Interestingly, mice from the C57Bl6N background were more resistant to noise induced hearing loss (Supplementary Figure S4) and the protocol of noise exposure needed to be adapted for comparable deafening (116dB SPL for C57Bl6/J and 120dB SPL for C57Bl6/N). In both p22^phox^ and NOX3 mouse strains, noise exposure led to a significant elevation of hearing thresholds within 24 h (Figures 3B,D; Supplementary Figure S5). The degree of hearing loss was comparable across mutants and wild-type animals and arose mostly at high frequencies, with about 20–40 dB SPL hearing loss between the frequencies of 16 and 32 kHz. Only in one frequency, at 32 kHz and only in the NOX3 mutant mice (Figure 3B), a statistically significant protection was observed compared to wild-type littermates at this time point. To address the more permanent threshold shift, ABR measurements were obtained 7 days following noise exposure (Figures 3C,E). At this time point, both mouse strains, p22^phox^ and NOX3, showed a partial but statistically significant recovery of hearing, in contrast to wild type littermates, where no signs of recovery were observed (Supplementary Figure S5).

### NOX3 Deficiency Prevents Noise-Induced Damage to the Neurosensory Cellular Structures in Cochlea

**FIGURE 4:**
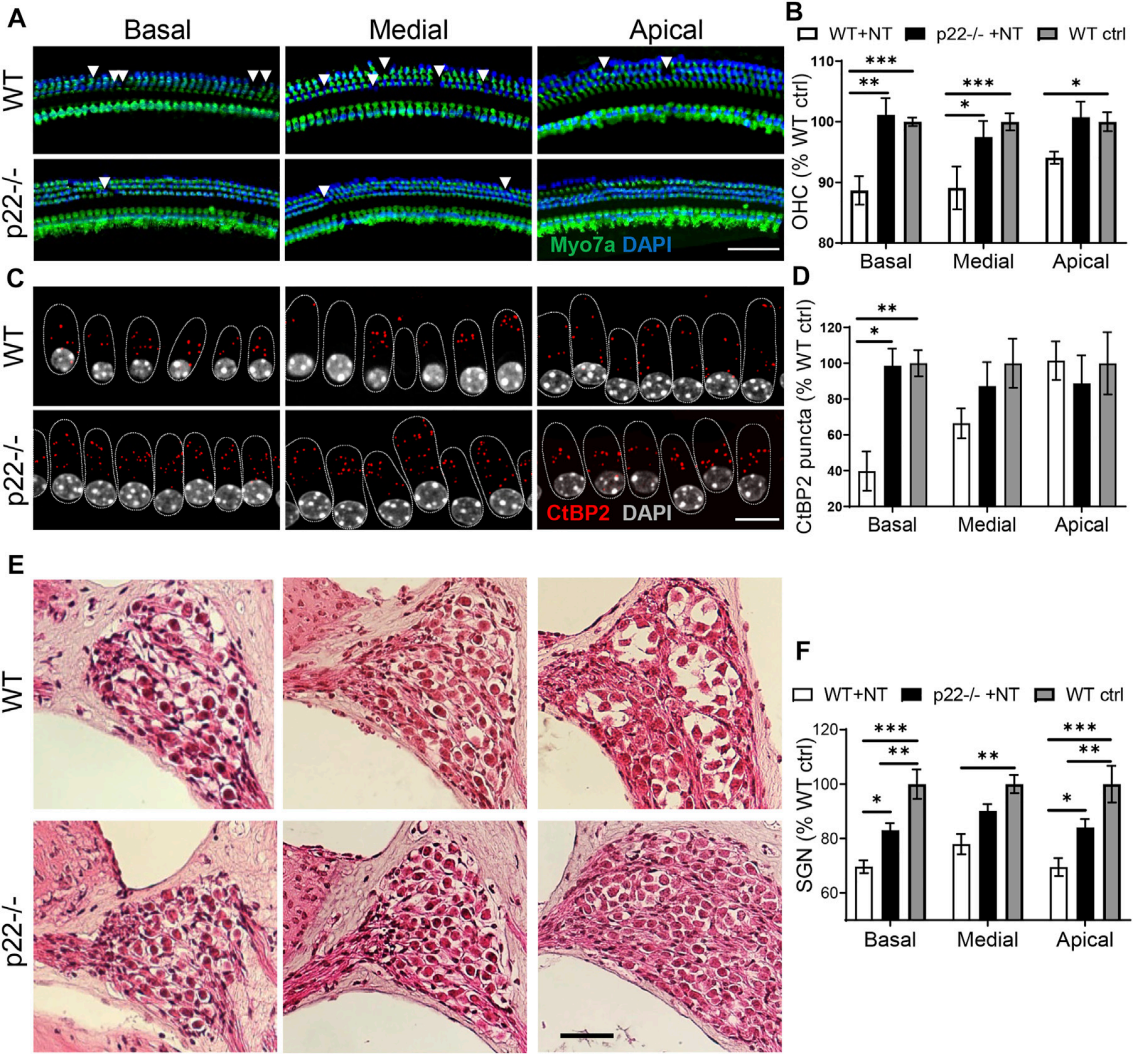
Cochlear histology of p22^phox^ knockout mice upon noise exposure. Seven days following the noise exposure, after the last audiogram determination, mice were sacrificed for cochlear histology. **(A)** Cytocochleogram showing the sensory epithelium in the three cochlear turns in WT (upper picture) and p22^phox^−/− littermates (lower picture). In green, the hair cell marker Myo7a and in blue, DAPI. Scale bar 20 µm. **(B)** Bar graph showing the outer hair cells number in the three cochlear turns of noise exposed WT and p22^phox^−/− littermates expressed relatively to WT non-exposed controls. *n* = 8 WT noise exposed (WT + NT); *n* = 7 p22^phox^−/− noise exposed (p22 + NT); *n* = 8 WT non-exposed controls (WT ctrl). **(C)** On the same samples, the number of synaptic ribbons between inner hair cells and spiral ganglion neurons was determined using CtBP2 immunostaining (red); upper panel WT and lower panel p22^phox^−/−. Scale bar: 10 µm. **(D)** Bar graph showing the number of ribbons/inner hair cell (IHC) in the 3 cochlear turns of WT and p22^phox^−/− littermates. The data are expressed relatively to non-exposed WT controls. **(E)** Representative mid modiolar hematoxylin and eosin staining showing the three cochlear turns of noise exposed WT (upper panel) and p22^phox^−/− (lower panel) littermates. **(F)** Bar graph showing the density of spiral ganglion neurons in the different parts of the cochlea of noise exposed WT and p22^phox^−/− littermates expressed relatively to WT controls. Scale bar 50 µm. *n* = 5 WT noise exposed (WT ctrl); *n* = 6 p22^phox^−/− noise exposed (p22 + NT); *n* = 6 WT controls (WT ctrl).

Animals were sacrificed for cochlear histology seven days after the noise over-exposure. In non-exposed control animals, consistent with the functional outcome (Supplementary Figure S5), comparable cochlear histology was obtained between genotypes (Supplementary Figure S6). However, the impact of noise trauma on the sensory epithelium was milder in both p22^phox^ (Figure 4) and NOX3 (data not shown) mouse strains, showing a statistically smaller loss of outer hair cells when compared to wild type animals (Figure 4B). In WT mice, noise exposure led to dramatic decrease in the number of auditory synapses, predominantly in the basal cochlear turn (Figures 4C,D). This was accompanied by a decrease in auditory neuron density in all cochlear turns (Figures 4E,F). Remarkably, p22^phox^ knockout and NOX3 mutant mice were both protected against noise induced auditory synaptopathy and auditory neuropathy, with markedly conserved auditory synapses and neuron integrity (Figures 4E,F). NOX3-mediated damages were predominantly observed in the basal and, to lesser extent, medial turn of the cochlea.

## Discussion

Oxidative stress is an underlying feature of several forms of acquired sensorineural hearing loss, including noise trauma (Henderson et al., 2006; Maulucci et al., 2014; Rousset et al., 2015). In the present study, we investigated the possible contribution of the ROS generating NADPH oxidase NOX3 in noise-induced sensorineural hearing loss using two different mice strains: the NOX3 mutant C57BL/6J-*NOX3* ^*het−4J*^/J and a constitutive p22^phox^ knockout (C57BL/N *Cyba* knockout), both deficient in NOX3 activity. Our results demonstrate a partial, but statistically significant protection against noise-induced hearing loss along with preserved cochlear cellular structures in both mouse strains. These findings are in line with previous studies on two other forms of acquired hearing loss, namely the drug-induced and age-associated hearing loss (Mukherjea et al., 2010; Kaur et al., 2016; Dhukhwa et al., 2019; Rousset et al., 2020; Mohri et al., 2021).

To the best of our knowledge, two previous studies investigated the effect of NOX on noise-induced hearing loss (Mohri et al., 2021; Lavinsky et al., 2015). The first study reported a small protective effect of NOX3 against noise-induced hearing loss but solely at a very specific frequency (8 kHz) (Lavinsky et al., 2015). In contrast, the study by Mohri et al. showed significant protection of NOX3-deficient mice (Mohri et al., 2021). We were not able to identify major differences in experimental protocols that could explain the discrepancies. Therefore, the role of NOX3 in the context of noise induced hearing loss remained to be clarified. Our study showed a significant protection of hearing at high frequencies in NOX3-deficient mice, however no frequency-specific protection at 8 kHz. In addition, the high frequency thresholds protection was validated in the second mouse strain with a loss of function mutation in the NOX3 subunit p22^phox^. Note also that the similarities of the protective effect of NOX3 deficiency and p22^phox^ deficiency suggest that there is not a major role of other p22^phox^-dependent NOX isoforms (i.e. NOX1, NOX2, and NOX4) in the pathophysiology of noise induced hearing loss. Furthermore, *Nox1* was not detectable in cochlear samples by real-time qPCR and neither *Nox2* nor *Nox4* were found at significant level in cochlear structures important for hearing, such as auditory neurons, organ of Corti, or stria vascularis, at least by using the RNAscope method (Figure 1; Supplementary Figure S1). Finally, noise overexposure primarily affects the high frequencies reflecting increased damage susceptibility of the basal cochlear turn. Interestingly, the tonotopic pattern of noise induced damages in the cochlea correlates with *Nox3* induction of expression [Figure 2, and reference (Son et al., 2012)] and is significantly mitigated upon Nox3 or p22^phox^ deletion.

To palliate the lack of specific antibodies against Nox3 (Diebold et al., 2019), Mohri et al. recently developed a mouse model with a reporter gene expressed upon Nox3 promoter (Mohri et al., 2021). *In situ* hybridization is another sensitive and reliable method to detect NOX mRNA (Moll et al., 2018; Rousset et al., 2020). Our data show important enrichment of *Nox3* mRNA in the Rosenthal canal area, whereas, the study by Mohri et al. shows rather scarce number of Nox3 positive auditory neurons. This apparent discrepancy could be explained by differences in sensitivity to detect *Nox3* at low levels. It is also possible that, since reporter gene knock-in with the *Nox3* reporter mouse leads to *Nox3* allele inactivation, putative Nox3 self-regulation loops are affected. Nevertheless, both the Nox3 reporter mouse and *in situ* hybridization methods were able to detect the increased transcription of *Nox3* following Noise exposure and upon ageing (Rousset et al., 2020).

In response to excessive noise, the kinetics of oxidative damage includes a rapid NADPH oxidation followed by lipid peroxidation, affecting membrane properties of cochlear hair cells and preceding metabolic impairment and cellular degeneration (Maulucci et al., 2014). In this context, the increase of NOX3 expression upon noise exposure is particularly interesting. Consistent with a previous report (Dhukhwa et al., 2019), we observed an increase in NOX3 expression within 24 h following the noise exposure, suggesting a possible time correlation with the increase of NADPH oxidation and lipid peroxidation. Whether NOX3 is directly responsible for NADPH oxidation as a result of its catalytic activity requires further experimental demonstration, as NADPH is also a major electron donor for antioxidant systems (Agledal et al., 2010). Furthermore, as suggested in our previous study on presbycusis, NOX-derived ROS may not only damage the neurosensory cells of the cochlea through oxidative stress but rather through indirect consequences of specific redox signaling (Rousset et al., 2020). The precise kinetics of NOX3 expression and its histological correlates remain therefore to be fully understood in the context of noise-induced hearing loss.

It is noteworthy to mention the difference in noise susceptibility between C57Bl6/J and C57Bl6/N genetic backgrounds (Supplementary Figure S4). As previously reported (Kendall and Schacht, 2014), C57Bl6/N mice were more resistant to noise insult than C57Bl6/J mice. In fact, C57Bl6/J mice present a deletion in the NNT (Nicotinamide Nucleotide transhydrogenase) gene resulting in a significantly decreased enzyme activity. Interestingly, this gene encodes an enzyme that uses energy from the mitochondrial proton gradient to produce high concentrations of NADPH, used for instance for free radical detoxification. It appears therefore likely that C57Bl6/J mice are more susceptible to oxidative damage, as induced by noise exposure *via* NOX3 activity.

NOX3-derived oxidants are essential for normal vestibular development (Paffenholz et al., 2004; Kiss et al., 2006; Nakano et al., 2008; Jones et al., 2010). However, its physiological relevance for the development and normal functioning of the cochlea remains unclear. We observed no difference in hearing thresholds and cochlear histology between NOX3-deficient mice and their wild-type littermates, corroborating the fact that NOX3 is not needed for a normal cochlear development and function, at least to a relevant extent. In a recent study, we have demonstrated that NOX3 is regulating the expression of genes involved in the auditory neuron excitatory pathway (Rousset et al., 2020). One could therefore propose NOX3 as a regulator of auditory neuron excitability in the auditory system, possibly tuning firing thresholds of auditory neurons, consistent with a previous proposed role for redox signaling in cortical neurons (Bothwell and Gillette, 2018). Further electrophysiological characterization of NOX3-deficient auditory neurons is needed to validate this hypothesis. In pathological conditions, including ageing (Rousset et al., 2020) cisplatin (Mukherjea et al., 2010) or noise overexposure, NOX3 may lead to neuron overactivation and excitotoxicity (Morton-Jones et al., 2008).

From a therapeutic perspective the observed temporal functional changes after noise trauma offer interesting opportunities: the inactivation of NOX3 seems to be functionally more relevant in the context of recovery from the insult at 7 days after the noise exposure compared to the immediate aftermath at day one. In a clinical context, patients with acute noise trauma (i.e. explosions and various other accidents) could be offered NOX3 inhibiting treatment in the hours following the insult and expect a better recovery. Whether the commonly used glucocorticoids for these situations today work through inhibition of NOX3, or whether a more specific NOX3 inhibition through pharmacological or molecular intervention is more effective remains to be investigated in the future. In absence of a bona fide NOX3 inhibitor, a nucleotide-based approach seems to be more promising (Rousset et al., 2019), at least based on the situation today.

In conclusion, our data demonstrates that NOX3 contributes significantly to noise-induced cochlear damage as previously demonstrated for two other forms of acquired forms of sensorineural hearing loss, namely age-related and cisplatin-induced hearing loss. NOX3 activity therefore seems to be a common molecular trigger influencing redox regulated neuronal activity, pathologically altered in different forms of acquired sensorineural hearing loss (Bothwell and Gillette, 2018; Wang and Puel, 2018; Rousset et al., 2020; Mohri et al., 2021). This corroborates the strong rationale to target NOX3 activity for prevention or treatment of noise-induced and other forms of acquired sensorineural hearing loss.

## Methods

### Animal Procedures

C57Bl6/J and C57Bl6/N mice sub-strains were employed during this study. *Nox3* mutant mice (C57BL/6J-Nox3^het−4J^/J, stock 005014) were purchased from Jackson laboratory, while p22^phox^ knockout (C57BL/N *Cyba* knockout) were generated in Prof. Ulla Knaus laboratory (Pircalabioru et al., 2016). Both mouse lines are constitutively deficient in functional NOX3 complex. In addition to NOX3 deficiency, p22^phox^ knockout mice are also devoid of functional NOX1, NOX2 and NOX4. Colonies were maintained at the animal facility of the University of Geneva through heterozygous x heterozygous breading, ensuring equal proportion of WT and NOX3 or p22^phox^ deficient littermates. All animal procedures inducing animal discomfort were performed under intraperitoneal (IP) ketamine (10%) and xylazine (5%) anesthesia (dose 10 μL/g). If necessary 10% ketamine solution was injected intramuscularly (dose 5 μL/g) to elongate the anesthesia. For the noise trauma, 6 weeks old mice were subjected to an 8–16 kHz noise band for 2 h at 116–120 dB SPL (Figure 3A), under anesthesia. At the end of the experiment (day 7), animals were sacrificed by cervical dislocation followed by decapitation. Cochlear samples were collected for histology evaluation or mRNA extraction.

### Auditory Brainstem Response

Hearing thresholds were tested by Auditory Brainstem Response (ABR) before noise exposure (pre-ABR), 24 h (D1) and 7 days (D7) after noise trauma. ABR recordings were performed conform previously described protocols (Rousset et al., 2020). Briefly, anesthetized animals were placed in a sound proof chamber (IAC Acoustics, Illinois IL, United States) upon a heating pad to maintain body temperature. Depth of anesthesia was tested every 30 min by the pedal withdrawal reflex. For the recordings, platinum electrodes were placed subcutaneously on the mouse forehead (+), on the mastoid of the recorded ear (−) and a reference electrode on the back. ABRs were recorded, following stimulation with 100 μs clicks or 3 m tone pipes (2.0–45.2 kHz at a resolution of 2 steps per octave). For all frequencies, they were recorded from 0 to 90 dB SPL in 3 dB steps. Electrical responses were averaged over 256 repetitions of stimulus pairs with alternating phase. Hearing thresholds were defined as the last sound pressure level with a conserved response pattern, identified by visual inspection of the averaged signal. For stimulus generation and recording of responses, a multi-function IO-Card (National Instruments, Austin TX, United States) and an IBM compatible computer were used. An integrated software package for stimulus generation and recording (Audiology_lab; Otoconsult, Frankfurt, Germany) was used. The sound pressure level was controlled with an attenuator and amplifier (Otoconsult, Frankfurt, Germany). Stimuli were delivered to the ear in a calibrated open system by a loudspeaker (AS04004PR-R, PUI Audio, Inc., Dayton, United States) placed 3 cm lateral to the animals’ pinna. The sound pressure was calibrated on-line prior to each measurement with a microphone probe system (Bruel and Kjaer 4191) placed near the animals’ ear. Recorded signals were amplified and bandpass filtered (80 dB; 0.2–3.0 kHz) using a filter/amplifier unit (Otoconsult, Frankfurt, Germany).

### Cochlea Histology

Following D7 ABR, anaesthetized mice were sacrificed. Temporal bones were isolated from the mice skull, in order to further dissect the auditory bulla. Dissected cochleae were placed in 4% paraformaldehyde overnight at room temperature. Cochleae were decalcified using USEDECALC solution (Medite commercial solution) under sonication for 48 h (Medite, Cat. No. 03-3300-00). After decalcification, both cochlea from the same animal were arranged for different protocols. While one cochlea was embedded in paraffin for cochlear morphology assessment (Hematoxilin-Eosin staining or RNAscope), the contralateral cochlea was micro-dissected to evaluate the sensory epithelium (cytocochleograms) as previously described (Rousset et al., 2020).

### Immunohistochemistry and Confocal Microscopy (Cytocochleograms)

The decalcified cochleae were dissected with microsurgical forceps under a binocular microscope as previously described (Rousset et al., 2020). Briefly, the bony shell was removed to expose the Organ of Corti (OC), followed by the removal of the stria vascularis and separation of the sensory epithelium from the spiral ganglion. The basal, middle and apical turns of the OC were respectively separated and transferred into 400 uL PBS solution in a 48 well plate. Samples were then permeabilized (3% Triton-X 100 in PBS 1X) for 30 min at room temperature and immersed in a blocking buffer, containing 2% bovine serum albumin (BSA) and 0.01% Triton-X 100 in PBS, for 30 min at room temperature. Explants were incubated overnight at 4°C with the primary antibodies anti-MyoVIIa (1:200, rabbit; Proteus, United States) and anti-Ctbp2 (1:200, monoclonal mouse, BD Bioscience) prepared in blocking buffer. On the following day, tissues were rinsed three times with PBS and incubated, for 2 h at room temperature, with the secondary antibodies anti-rabbit Alexa Fluor 488 (1:500; Invitrogen, United States) and goat anti-mouse Alexa 555 (1:500; Life Technologies) diluted in blocking buffer. Explants were washed 3 times with PBS and mounted on a glass slide with Fluoroshield containing DAPI (Sigmaaldrich, United States). The labelled cells were visualized with a confocal laser-scanning microscope (Zeiss LSM700) equipped with a CCD camera (Leica Microsystems) employing the Plan-Neofluar 20X/0.50 and Plan-Apochromat 63X/1.4 (oil) objectives. Hair cell and ribbons quantification was performed using ImageJ software.

### Mid Modiolar Preparations

Following standardized protocols, decalcified cochleae were sequentially dehydrated and embedded in paraffin. Mid-modiolar cuts of 5 µm were processed and loaded onto gelatin-coated slides. Adjacent mid-modiolar cuts were employed on hematoxylin/eosin and RNAscope protocols (see sections below).

### Hematoxylin Eosin Staining

Harris’ hematoxylin/eosin staining was performed on 5 non-consecutive slides of each cochlea including all cochlear turns. Mid-modiolar paraffin slides were re-hydrated with successive xylene and alcohol baths, then exposed to Hematoxylin for 5 min, rinsed with H_2_O and briefly submerged in an alcohol and HCl bath, and finally washed with alcohol 70%. Following Hematoxylin staining, slides were briefly (2–3 s) exposed to the eosin reagent. After staining the slides were dehydrated with alcohol and xylol in successive bath steps, and mounted with the commercial Eukitt mounting medium. Images were obtained with a Zeiss Axioskop 2 plus microscope. Spiral ganglia density was reported and assessed on the resulting images using ImageJ software.

### Quantitative Analysis of Cochlea Morphology

Hematoxylin-eosin mid-modiolar cuts images were analyzed on the open source software ImageJ to determine the spiral ganglion neurons density. SGN nuclear quantification was performed and normalized to the Rosenthal’s canal area (in mm^2^) within all cochlear turns (apical, medial and basal). Five non-consecutive sections were evaluated for each turn and the densities were averaged. Similarly, cytocochleogram images were employed to determine the number of synapsis/inner hair cell and outer hair cell viability. For synaptic ribbons quantification, images were recorded with a 63X confocal objective, generating 10-15 um Z stack images (0.7 um steps). The resulting file was projected into a single plane to ensure accurate quantification of the ribbons distributed along the *Z* axis. For each cochlear turn, two different segments of 10–15 inner hair cells were assessed to obtain an average number of ribbon synapses. In the same way, the hair cell viability (cytocochleogram) was evaluated in two representative areas of 100 µm and the survival rate was averaged for each cochlear section (apical, medial and basal). Both Inner hair cells (IHC) and outer hair cells (OHC) were recorded with a 20X confocal objective. The open source ImageJ program was used for the image analysis.

### Cochlea RNA Extraction

To avoid RNA degradation, cochleae were quickly dissected in cold PBS, removing remaining blood and surrounding tissues, immediately frozen in liquid nitrogen and kept at -80°C for further RNA extraction as previously described (Rousset et al., 2020). An adapted protocol was employed for the RNA extraction [adapted from (Vikhe Patil et al., 2015)], based on the Qiagen RNeasy Micro kit, replacing the lysis and homogenization steps. Samples were physically homogenized with clean steel beads and tissueLyser (Qiagen) for 30 s at 30 rpm. Trizol (750 uL) was added to the lysate, precipitating the sample DNA and RNA. After adding 150 ul chloroform, (shaking, resting and centrifuging step) the aqueous phase was collected and followed the purification protocol described on the commercial kit.

### Real Time Quantitative Polymerase Chain Reaction

Following RNA purification and tritation, 500 ng of RNA were prepared for cDNA synthesis using the Takara PrimeScript RT reagent Kit, following manufacturer’s instruction [see also (Rousset et al., 2020)]. Real-time PCR was performed using SYBR green assay on a 7900HT SDS system from ABI. The efficiency of each primer was verified with serial dilutions of cDNA. Relative expression levels were calculated by normalization to the geometric mean of the three house-keeping genes (*Eef1a*, *Tubb* and *Actb*). The highest normalized relative quantity was designated as a value of 1.0. Fold changes were calculated from the quotient of means of these RNA normalized quantities and reported as ±SEM. Sequences of the primers used are provided in Supplementary Table S1.

### RNAscope^®^ *in situ* Hybridization (ACDbio, Bio-Techne, Minneapolis, Minnesota, United States)

Mid-modiolar cuts (5 µm) obtained from decalcified cochleae (see section above - mid modiolar preparations) were loaded onto gelatin-coated slides and followed RNAscope probe hybridization. RNAscope 2.5 HD Assay–BROWN assay (Bio-techne, Cat. No. 322310) or Fluorescent multiplex assay (Bio-techne, Cat. No. 320850) was performed according to manufacturer’s protocol. Selected paraffin sections were hybridized with the probes Mm-NOX3-C1 (Bio-techne, Cat. No. 481989), Mm-Cybb (Bio-techne, Cat. No. 403381), Mm-NOX4 (Bio-techne, Cat. No. 457261), Mm-Ppib-C1 (Bio-techne, Cat. No. 313911) as positive control and DapB-C1 (Bio-techne, Cat. No. 310043) as negative control at 40°C for 2 h and revealed with TSA Opal570 (Perkin Elmer, Cat. No. FP1488001KT). For BROWN assay, Mayer hematoxyline staining (30 s) was employed as counterstaining agent, whereas DAPI and *β*-III tubulin (1/1000, rabbit, Abcam, ab52623) immunostaining were used for Fluorescent multiplex assay. Finally, the slides were dehydrated, cleared and mounted with Tissue-Tek^®^ Glas™ (Sakura, Cat. No. 1408). Samples were visualized with a confocal laser-scanning microscope (Zeiss LSM700) equipped with a CCD camera (Leica Microsystems) employing the Plan-Neofluar 20X/0.50 and Plan-Apochromat 63X/1.4 (Oil) objectives. Pictures were analyzed using the open source software ImageJ.

### RNAscope^®^ Signal Quantification

RNAscope samples were analyzed and quantified employing the open-source software FIJI. RNAscope signal quantification was automatized employing FIJI’s macro functionality together with Ilastik software as segmentation tool. Multi z-stack images were recorded employing a Axiocam Fluo microscope with a 40X EC Plan-Apochromat objective. Multiple tiles comprising the region of interest were acquired and merged employing Zeiss commercial software to generate a single image.

Employing FIJI macro software, the resulting z-stack images were projected into a single plane and divided into 2 channels. Blue channel (DAPI, nucleus) was processed accordingly to reduce background noise. The red channel (RNAscope signal) followed a Richardson-Lucy deconvolution (DeconvolutionLab2 plugin, 45 iterations) followed by proper processing (background subtraction). Appropriated PSF was provided during each image deconvolution, employing FIJI’s plugin “PSF generator” (macro automatized). The deconvoluted red channel was then segmented following a previously trained Ilastik model, and transformed to a binary image with Huang’s approach. Proper morphometric operators were applied and the RNAscope signal was quantified following FIJI’s “Analyze particle” command (1.5-Infinity pixel).

The designed macro then normalized the number of dots quantified on the previous section (RNAscope signal, red channel) to the area of interest (except for the Organ of Corti, whose values are absolute). In order to proceed with the normalization, we manually determine the area of interest (Rosenthal canal or stria vascularis) following each image blue channel. The results were represented as RNAscope dots/mm2 (except for the Organ of Corti). RGB images were automatically generated during the macro processing, allowing the user to verify the absence of anomalies during the process.

### Statistics

Real time qPCR data were analyzed using One-way ANOVA followed by Dunnett’s multiple comparison test. Other datasets were analyzed using Two-way ANOVA followed by Bonferroni multiple comparison test. GraphPad Prism software (version 8.4.3) was used. Values with *p* < 0.05 was considered as statistically significant. *p < 0.05, **p < 0.01, ***p < 0.005, ****p < 0.0005.

### Study Approval

All procedures were approved by the local veterinary office and the Commission for Animal experimentation of the Canton of Geneva, Switzerland, authorization number GE-28-18.

## Data Availability

The raw data supporting the conclusion of this article will be made available by the authors, without undue reservation.

## Abbreviations

NIHL, Noise-Induced Hearing Loss; NOX, NADPH oxidase; OC, Organ of Corti; ROS, Reactive Oxygen Species; SGN, Spiral Ganglion Neurons; SV, Stria Vascularis.
