# Supplementary material for: NADPH Oxidase 3 Deficiency Protects From Noise-Induced Sensorineural Hearing Loss
Source: Front Cell Dev Biol. 2022 Feb 22;10:832314. doi: 10.3389/fcell.2022.832314 (PMC8902251; doi:10.3389/fcell.2022.832314)
Supplement: Supplementary file 1 [file DataSheet1.PDF]

Supplementary Figure 1

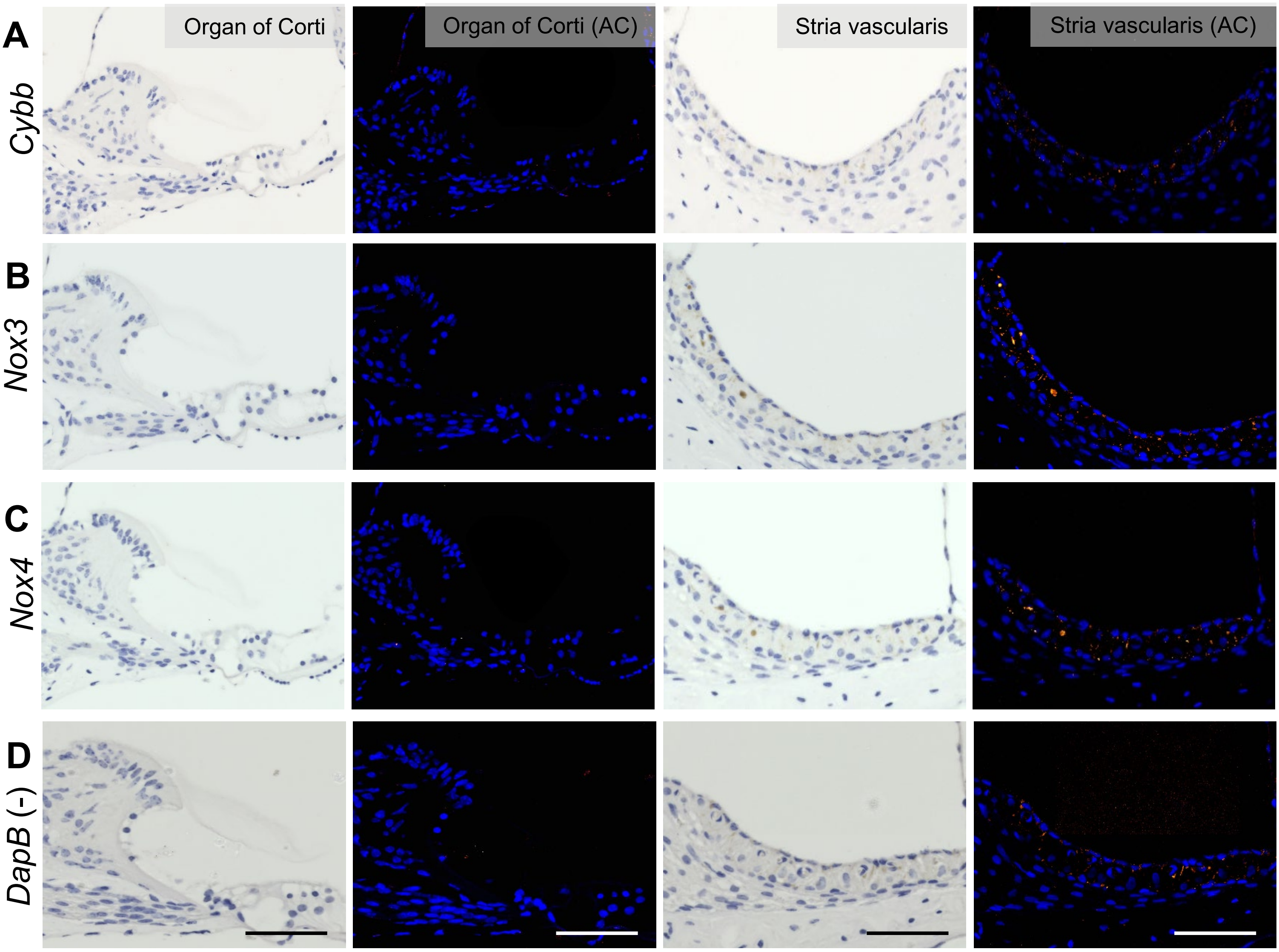

Supplementary Figure 1. Pattern of NOX expression in the mouse organ of Corti and stria vascularis. RNAscope *in situ* hybridization of *Cybb* (Nox2) (A), *Nox3* (B) and *Nox4* (C) in mouse organ of Corti and stria vascularis. Dihydrodipicolinate reductase (*Dapb*) expressed in the bacteria E. Coli was used as negative control (D). Scale bar 50µm. Each picture was duplicated with artificial color (blue / magenta) in order to facilitate RNAscope signal visualization, revealing little *Nox3* and *Nox4* expression in the stria vascularis. No NOX expression was detected in the Organ of Corti.

Supplementary Figure. 2

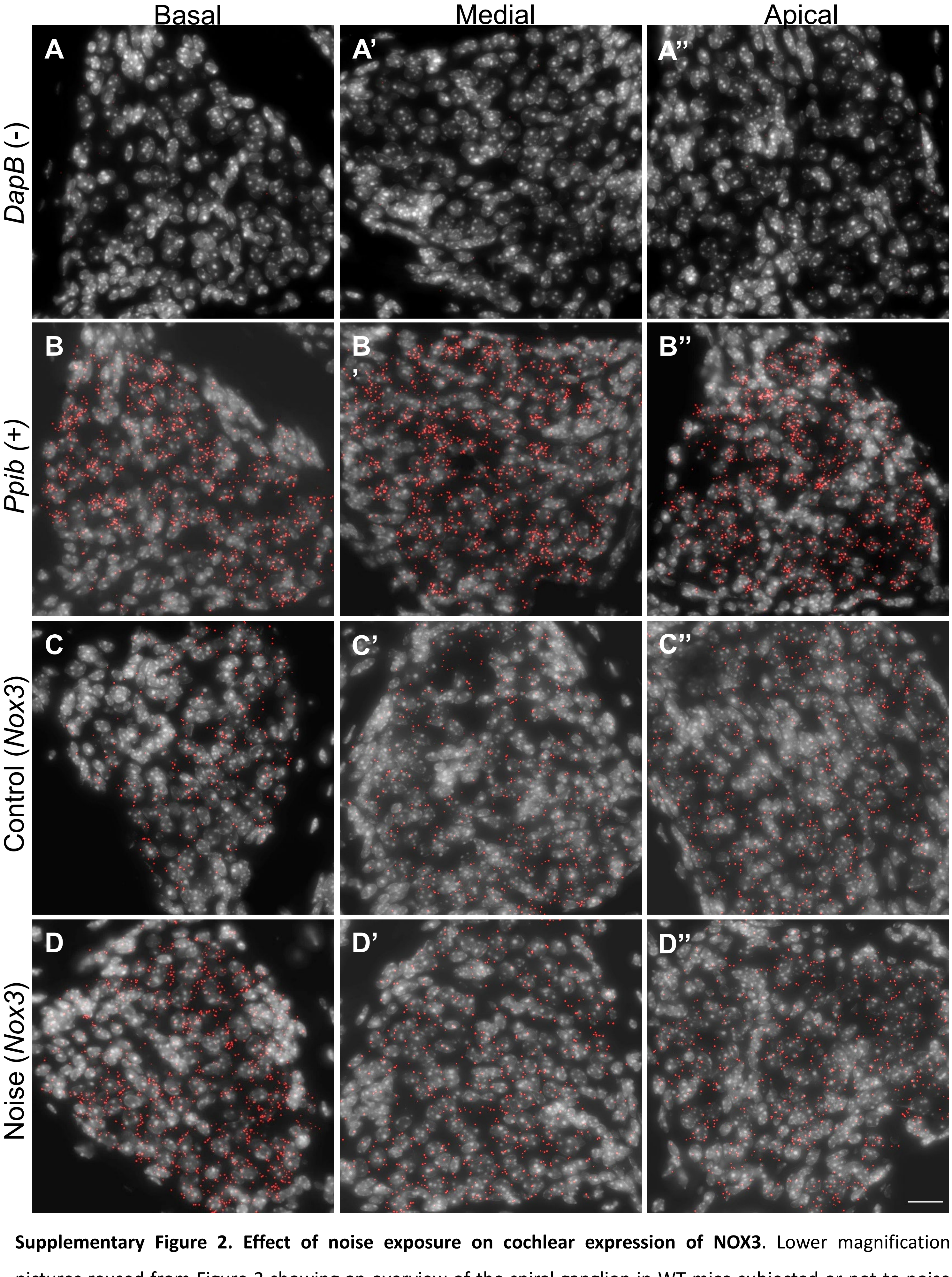

Supplementary Figure 2. Effect of noise exposure on cochlear expression of NOX3. Lower magnification pictures reused from Figure 2 showing an overview of the spiral ganglion in WT mice subjected or not to noise exposure. Scale bar = 20µm. To facilitate signal visualization under low magnification, the RNAscope signal was binarized and enhanced employing Huang formula on ImageJ. For detailed legend, please refer to Figure 2.

Supplementary Figure 3

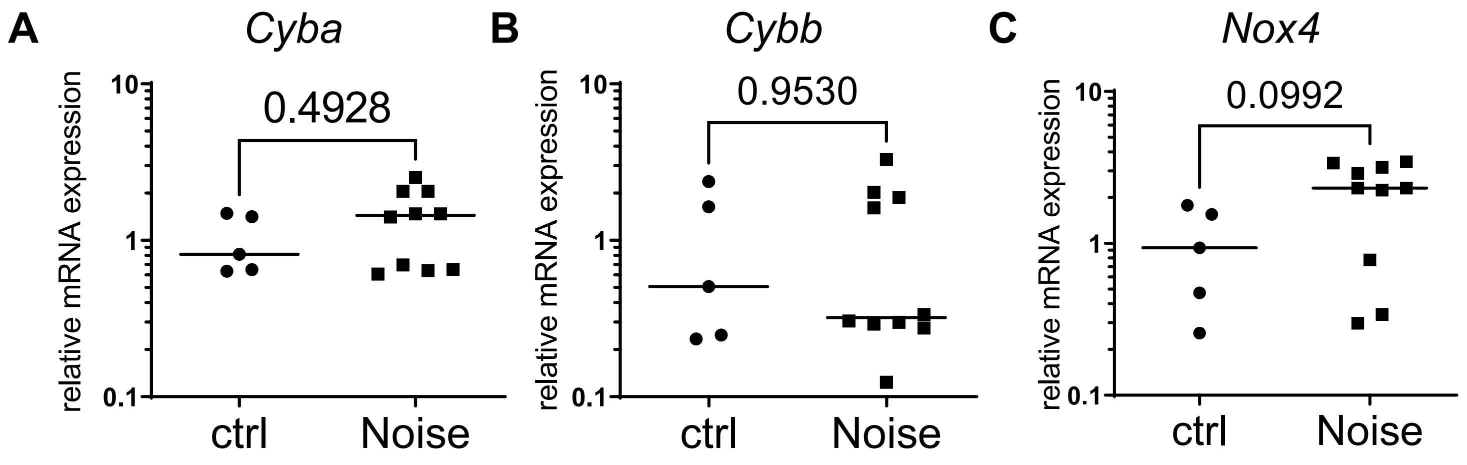

Supplementary Figure 3. Effect of noise exposure on cochlear expression of NOX. C57Bl6/J mice were exposed to an 8-16kHz 116dB SPL noise band for 2h. Cochleae were harvested 24h following noise exposure for Real time qPCR of *Cyba* (p22^{phox}) (A), *Cybb (*Nox2*)* (B) and *Nox4* (C) expression. Control non-exposed: n=5 animals; Noise exposed cochleae n=10 animals. No statistically significant changes could be detected following noise exposure with Mann-Whitney test.

Supplementary Figure 4

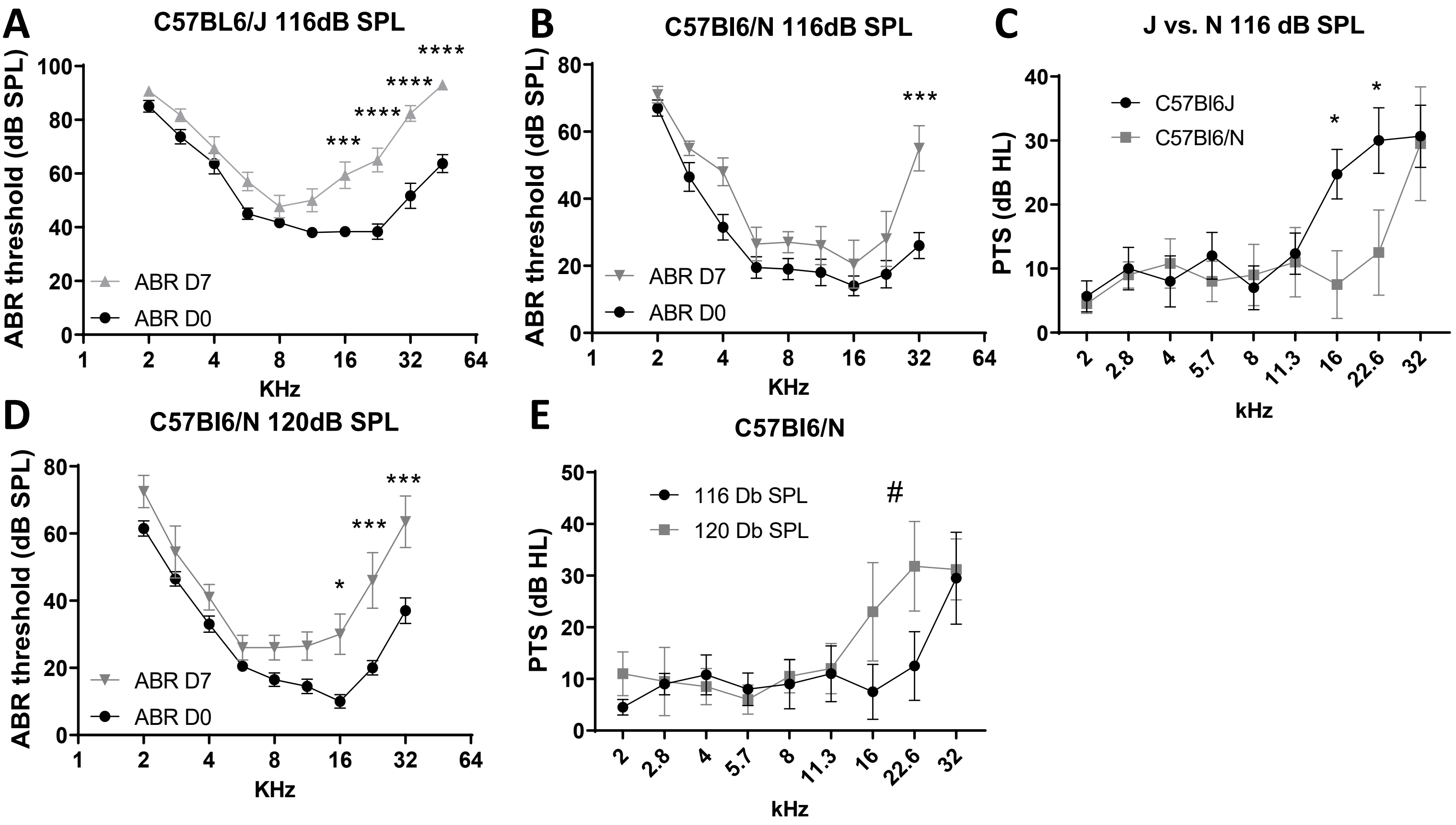

Supplementary Figure 4. Noise-deafening procedures in C57Bl6/J and C57Bl6/N. Audiograms of C57Bl6/J (A) and C57Bl6/N (B) before and after 2h exposure to a noise band 8-16kHz at 116dB SPL. C) Permanent threshold shifts as obtained 7 days following noise exposure shows increased susceptibility to noise in C57Bl6/J at 16 and 22 kHz. D) To generate significant noise deafening in C57Bl6/N the level of noise exposure was increased from 116dB SPL to 120 dB SPL. E) Comparison of permanent threshold shifts in C57Bl6/N mice following 116- or 120-dB SPL noise exposure. n=6 C57Bl6/N and 6 C57Bl6/J.

Supplementary Figure 5

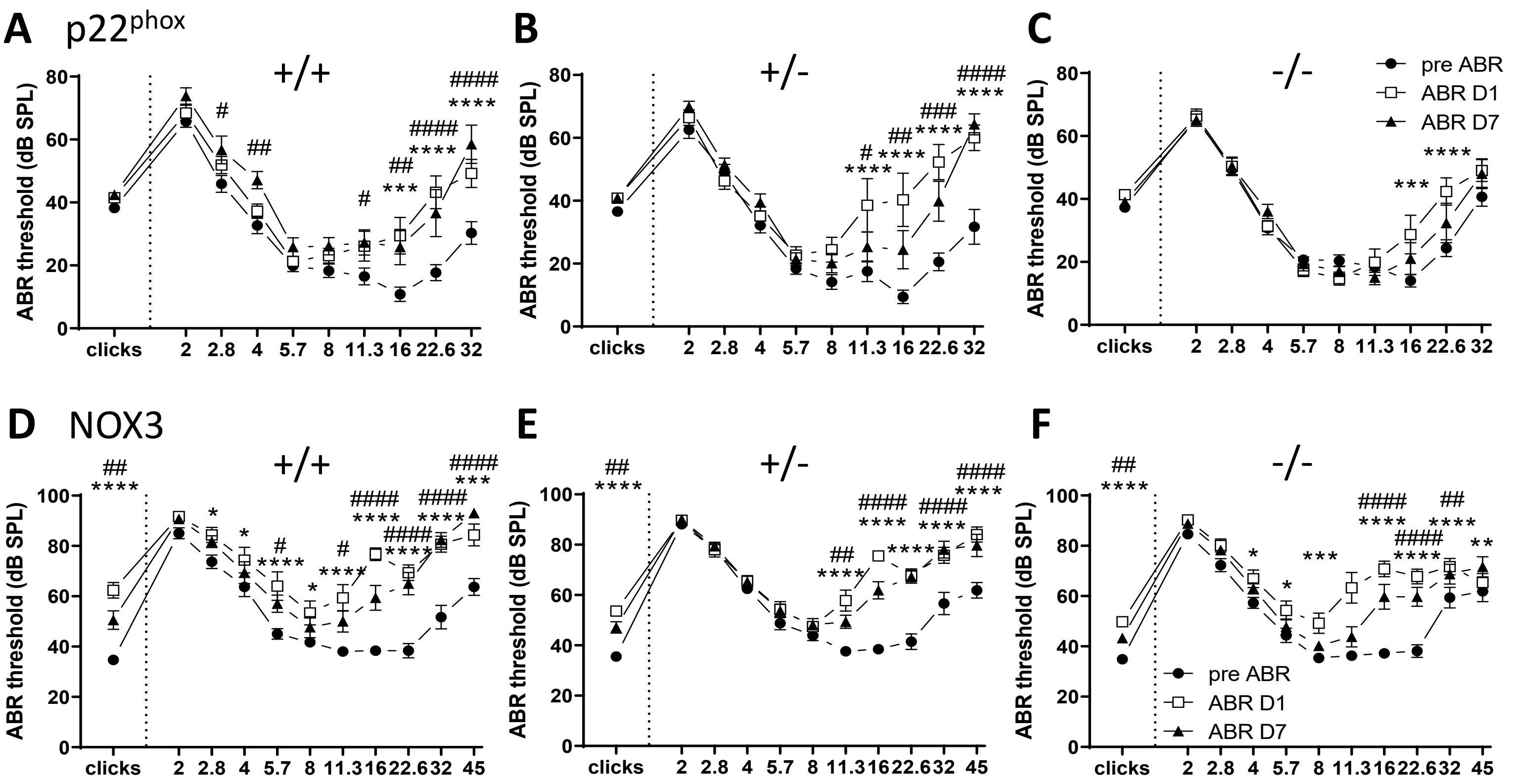

Supplementary Figure 5. Full audiograms of NOX3 and $p22^{phox}$ deficient mice. Full audiograms of Nox3 and $p22^{phox}$ deficient and their WT and heterozygous littermates. A-C) Audiogram of $p22^{phox}$ knockout mice as recorded before (pre ABR), one day (D1) and seven days (D7) following noise exposure. A) $p22^{phox}$+/+; B) $p22^{phox}$+/- and C) $p22^{phox}$-/-. D-E) Audiogram of NOX3 mutant mice as recorded before (pre ABR), one day (D1) and seven days (D7) following noise exposure. D) *Nox3*+/+; E) *Nox3*+/- and F) *Nox3*-/-. n=9 *Nox3*+/+; 11 *Nox3*+/-; 10 *Nox3*-/-; D-E) n=12 *Cyba*+/+; 7 *Cyba* +/-; 13 *Cyba* -/-. * $p<0.05$, ** $p<0.01$, ***; $p<0.005$, ****$p < 0.0005$ D0 vs. D7; # $p<0.05$, ## $p<0.01$, ### $p<0.005$, ####$p < 0.0005$ D0 vs. D1. The data for WT, Nox3-/- and $p22^{phox}$-/- are reused from Figure 3.

Supplementary Figure 6

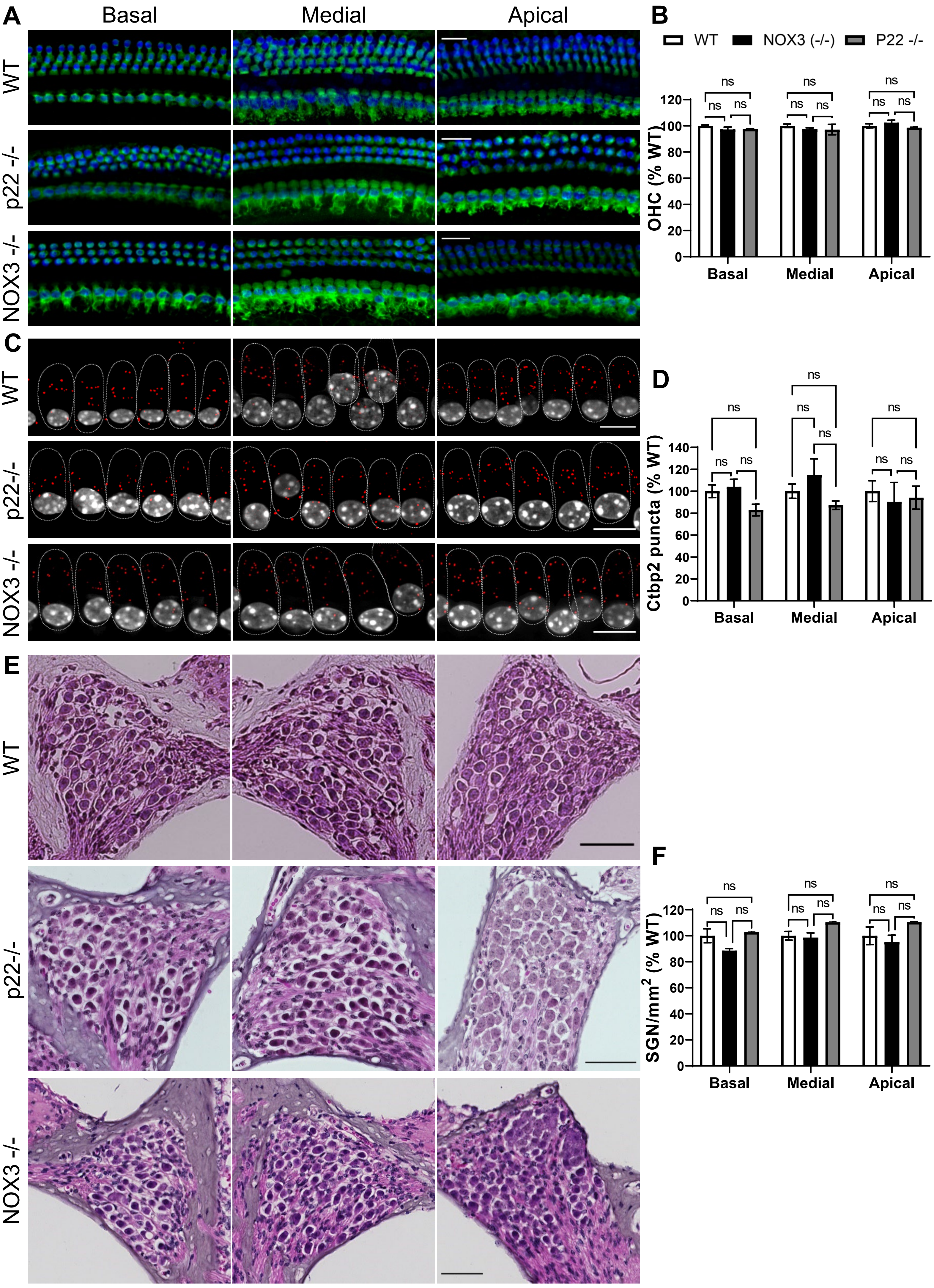

Supplementary Figure 6. Cochlear histology of NOX3, p22^{phox} and WT control mice without noise exposure. 6-8 weeks old mice from NOX3, p22^{phox} and WT genetic background were sacrificed for control cochlear histology. A) Cytocochleogram showing the sensory epithelium in the three cochlear turn in WT (upper picture) p22^{phox}-/- (middle) and NOX3 -/- (lower pictures) animals. In green, the hair cell marker Myo7a and in blue, DAPI. Scale bar 20µm. B) Bar graph showing the outer hair cells number in the three cochlear turn of NOX3 and p22^{phox}-/- littermates relatively to WT animals (100%). n = 5 WT; n = 4 p22^{phox}−/−; 4 NOX3 controls. C) On the same samples, the number of synaptic ribbons between inner hair cells and spiral ganglion neurons was determined using CtBP2 immunostaining (red); upper panel WT, middle panel p22^{phox} -/- and lower panel NOX3 -/-. Scale bar: 10 µm. D) Bar graph showing the number of ribbons/inner hair cell (IHC) in the 3 cochlear turns of NOX3 and p22^{phox}-/- animals relatively to WT (100%). E) Representative mid modiolar hematoxylin and eosin staining showing the three cochlear turns of noise exposed WT (upper panel) middle panel p22^{phox} -/- and lower panel NOX3 -/- animals. F) Bar graph showing the density of spiral ganglion neurons (SGN)/mm^2 in the different parts of the cochlea of NOX3 and p22^{phox} -/- animals expressed relatively to WT littermates (100%). Scale bar 50µm. n = 4 NOX3 -/- ; n = 2 p22^{phox}−/−; n = 6 WT controls.

Supplementary table 1: List of qPCR primers used in the study.

Gene (Mm)	Primer forward 5'-3'	Primer reverse 5'-3'
Eef1A1	TCCACTTGGTCGCTTTGCT	CTTCTTGTCCACAGCTTTGATGA
Tubb	GCAGTGCGGCAACCAGAT	AGTGGGATCAATGCCATGCT
Actin β	CTAAGGCCAACCGTGAAAAGAT	CACAGCCTGGATGGCTACGT
***Cyba* (p22^{phox})**	TGGACGTTTCACACAGTGGT	TGGACCCCTTTTTCCTCTTT
Nox1	CCCAGCAGAAGGTCGTGATT	GCTAAAGCCTCGCTTCCTCAT
***Cybb* (Nox2)**	CAGGAACCTCACTTTCCATAAGAT	AACGTTGAAGAGATGTGCAATTGT
Nox3	CGACGAATTCAAGCAGATTGC	AAGAGTCTTTGACATGGCTTTGG
Nox4	CCGGACAGTCCTGGCTTATCT	TGCTTTTATCCAACAATCTTCTTGTT

Supplementary table 2: List of primary and secondary antibodies.

Antibody	supplier	reference	dilution
TUJ-1	Biolegend	802001	1/2000
Ctbp2	BD Biosciences	15886079	1/200
Myo7a	Proteus Biosciences	25-6790	1/200
Alexa Fluor anti rabbit 555	Invitrogen	A31572	1/1000
Alexa Fluor anti mouse 555	Invitrogen	A31570	1/500
Alexa Fluor anti rabbit 488	Invitrogen	A21206	1/500

Supplementary table 3: List of reagents.

name	supplier	reference
Triton X-100	Sigma	X100
PBS	Sigma	D1408
Fluoroshield	Sigma	F6057
RNeasy Microkit	Qiagen	74004
Trizol	Life Technologies	15596018
Bovine serum albumine	Sigma	A3912
Hematoxyline Harris	Sigma	HHS16
Eosin	Sigma	HT110116
Eukitt	Sigma	03989
